# A Retrospective Comparison of Clinical and Laboratory Aspects of Patients With COVID-19-Related Acute Respiratory Distress Syndrome (ARDS) and Non-COVID-19-Related ARDS

**DOI:** 10.7759/cureus.63794

**Published:** 2024-07-04

**Authors:** Nazan Yıldız, Ebru Kaya, Ayca Sultan Sahin

**Affiliations:** 1 Anesthesiology and Reanimation, Kırklareli Training and Research Hospital, Kırklareli, TUR; 2 Intensive Care Unit, Sağlık Bilimleri Üniversitesi Kanuni Sultan Suleyman Training and Research Hospital, İstanbul, TUR; 3 Anesthesiology and Reanimation, Sağlık Bilimleri Üniversitesi Kanuni Sultan Suleyman Training and Research Hospital, İstanbul, TUR

**Keywords:** intensive care unit, non-covid-associated ards, covid-19 pneumonia, covid-19 associated ards, ards

## Abstract

Aim/Objective: This study investigated demographic characteristics, hemodynamic values, respiratory datas, laboratory values ​​such as biochemistry and blood gas, and treatment approaches of coronavirus disease 2019 (COVID-19)-related and non-COVID-19-related acute respiratory distress syndrome (ARDS) patients hospitalized in the intensive care unit (ICU).

Background: Determining the differences and similarities between COVID-19-related ARDS (CARDS) patients and non-COVID-19-related ARDS (NCARDS) patients will be useful to better understand these two diseases.

Materials and methods: A total of 32 NCARDS patients who were followed and treated in the ICU for various reasons between January 2015 and December 2020 and 32 CARDS patients who were followed and treated in the ICU for various reasons between March 2020 and December 2020 were examined retrospectively. Age, gender, comorbidities, Glasgow Coma Scale (GCS), Acute Physiology and Chronic Health Evaluation (APACHE) II Score, blood pressure, heart rate, saturation, laboratory results, arterial blood gas (ABG) values, length of stay in the ICU, intubation, the number of days till the patient was extubated, the treatments applied, admission to the service, and mortality were evaluated.

Results: In the comparison between the two groups, the demographic data of the patients, the number of days intubated and extubated, APACHE II scores, and ICU length of stay were not statistically different. Values of positive end-expiratory pressure (PEEP), first hospitalization GCS, first hospitalization hemoglobin (Hgb), albumin at first admission, alanine aminotransferase (ALT) at first admission, and steroid use were found to be significantly different in patients with CARDS (p < 0.001). The median of PEEP values (p = 0.04), first admission GCS values (p = 0.04), first admission Hgb values (p = 0.005), albumin values at the first admission (p = 0.03), ALT values (p = 0.03), and the rate of steroid use (p = 0.001) of CARDS patients were significantly higher than those of NCARDS patients. The median of the first hospitalization heart rate values (p = 0.009), first hospitalization saturation values (p = 0.001), and first admission neutrophil values (p = 0.03) in NCARDS patients were significantly higher than that of CARDS patients. There was no significant difference between the two groups in terms of mortality, sedation use, inotropic support, C-reactive protein (CRP), and procalcitonin values.

Conclusions: CARDS and NCARDS have clinical and laboratory similarities and differences. Therefore, there should be differences in our follow-up and treatment approach to these two disease groups.

## Introduction

The World Health Organization declared coronavirus disease 2019 (COVID-19) caused by the severe acute respiratory syndrome coronavirus 2 (SARS-CoV-2) virus, a significant threat to international health. COVID-19 primarily affects the respiratory system and can rapidly progress to acute respiratory distress syndrome (ARDS) in some patients. These patients have a high admission rate to intensive care units (ICUs) and a high mortality rate, which is closely associated with ARDS. While several studies have reported on the clinical characteristics of COVID-19, our understanding of it remains limited [1].

COVID-19 has emerged as a major public health problem, with a worldwide death toll of several hundred thousand in the first months of the epidemic. Between 14% and 26% of patients hospitalized with COVID-19 require admission to ICUs, and preliminary reports indicate that ICU mortality is high in patients receiving invasive mechanical ventilation [2].

It has long been known that patients with ARDS have markedly different clinical presentations. The Berlin definition does not include a threshold value for respiratory compliance as a diagnostic criterion for ARDS [1]. Furthermore, the clinical characteristics, ventilator management, and patient outcomes of patients with COVID-19-associated ARDS (CARDS) are not well defined [3].

Can we consider all cases of acute respiratory failure associated with COVID-19 as ARDS? The answer to this question is likely “no”. Based on current reports and our experience in managing CARDS patients, there are significant differences between CARDS and ARDS caused by factors other than those defined by the Berlin criteria. Consequently, there are differences in their treatment approaches [1].

## Materials and methods

This study was conducted following ethical approval from the Istanbul Ministry of Health University Kanuni Sultan Süleyman Training and Research Hospital Clinical Research Ethics Committee, with approval obtained in January 2021 under meeting and decision number KAEK/2021.01.26.

The medical records and computer core system data of 32 CARDS patients who were admitted to the Anesthesiology and Reanimation Unit of Istanbul Ministry of Health University Kanuni Sultan Süleyman Training and Research Hospital, Türkiye, between March 2020 and December 2020 and 32 non-COVID-19-related ARDS (NCARDS) patients who were admitted to the same hospital between January 2015 and December 2020 were retrospectively scanned and examined.

Inclusion and exclusion criteria

Inclusion criteria for the study were as follows: patients between the ages of 18 and 80, patients diagnosed with ARDS according to the Berlin criteria, patients who had COVID-19 pneumonia, and patients who were monitored in the COVID-19 ICU.

Exclusion criteria for the study were as follows: patients outside the age range of 18-80, patients who did not meet the Berlin diagnostic criteria for ARDS, patients who did not have COVID-19 pneumonia, patients in the postoperative period, and patients with an ICU stay duration of less than 24 hours.

Data collection

From the patients' files and the hospital computer system, age, gender, history of comorbidities, number of days hospitalized, number of days on a ventilator, number of days intubated, positive end-expiratory pressure (PEEP) (PEEP titration was applied to the patients based on the PEEP levels that could be applied according to the fraction of inspired oxygen (FiO2) value recommended by the ARDS Clinical Network study), FiO2, blood pressure, average pressure, fever, pulse, laboratory tests, Acute Physiology and Chronic Health Evaluation (APACHE) II scoring, treatments used (medical drugs, prone position, hemodiafiltration), and discharge or mortality status were collected and analyzed. It is to be noted that prone positioning was to be applied for more than 12 hours per day in moderate-severe ARDS and CARDS cases (partial pressure of oxygen (PaO2)/FiO2<150), as recommended by our Ministry of Health.

Statistical analyses

Statistical analyses were performed using IBM SPSS Statistics for Windows, Version 19.0 (Released 2010; IBM Corp., Armonk, New York, United States). Descriptive criteria, Mean and standard deviation, median and min-max values ​​are presented as percentage distribution. The suitability of the data for normal distribution was checked with the Kolmogorov-Smirnov test. Chi-square analysis was used to compare the distributions between groups, the Student-t test was used to compare the means in independent groups when parametric conditions were met, the Mann-Whitney U test was used when parametric conditions were not met, and the Wilcoxon Signed Rank test was used if parametric conditions were not met in dependent groups. The significance level was taken as p<0.05.

## Results

This study was conducted based on the data of CARDS and NCARDS patients admitted to the Anesthesiology and Reanimation Intensive Care Unit of Istanbul Ministry of Health University Kanuni Sultan Süleyman Training and Research Hospital within a five-year period between January 2015 and December 2020.

Of the 64 patients examined, 32 were CARDS patients and 32 were NCARDS patients. Among the CARDS patients, 15 (46.9%) were female and 17 (53.1%) were male. Among the NCARDS patients, 16 (50%) were female and 16 (50%) were male (Table 1).

**Table 1:** Comparison of COVID-19-related ARDS patients and non-COVID-19-related ARDS patients’ gender and comorbidities ARDS: acute respiratory distress syndrome; COVID-19: coronavirus disease 2019

	COVID-19-related ARDS patients		Non-COVID-19-related ARDS patients		p-value
	Number of Patients	Percentage	Number of Patients	Percentage	
Sex					
Male	17	53.1	16	50.0	0.80
Female	15	46.9	16	50.0	
Comorbidity					
-	4	12.5	13	40.0	0.113
+	28	87.5	19	60.0	

In CARDS-diagnosed patients, 87.5% had at least one comorbidity. This rate was 60% in NCARDS patients. According to the Chi-squared test, there was no statistically significant difference in terms of comorbidity between the two groups (p > 0.05). Four (12.5%) CARDS patients had no comorbidities, while 28 patients (87.5%) had at least one comorbidity. Among the comorbidities of patients monitored in the ICU, some were related to conditions such as hypertension, diabetes mellitus (DM), asthma, coronary artery disease (CAD), and chronic kidney disease (CKD). Additionally, three patients had pregnancy as a comorbidity. However, 13 (40%) of the NCARDS patients had no comorbidities, while 19 patients (60%) had at least one comorbidity. Among the comorbidities of NCARDS patients monitored in the ICU, some were related to conditions such as hypertension, DM, asthma, CAD, and CKD. There were no pregnant patients in this group.

The mean age of CARDS patients was 58.3 years. For NCARDS patients, the mean age was 49.7 years. According to the Wilcoxon signed-rank test, there was no statistically significant difference (p = 0.06) (Table 2).

**Table 2:** Comparison of the ages of COVID-19-related ARDS patients and non-COVID-19-related ARDS patients IQR: interquartile range; ARDS: acute respiratory distress syndrome; COVID-19: coronavirus disease 2019 ^a ^Wilcoxon signed-rank test

	COVID-19-related ARDS patients		Non-COVID-19-related ARDS patients		p-value
Age^a^	Mean	58.3	Mean	49.7	0.06
	Median	62.0	Median	47.5	
	Standard Deviation	15.4	Standard Deviation	20.6	
	IQR	22	IQR	36	

It was determined that 71.9% of CARDS patients used corticosteroids (methylprednisolone) at some stage of treatment. In contrast, only 31.3% of NCARDS patients used corticosteroids at some stage of treatment. According to the chi-squared test, the need for corticosteroid use in CARDS patients was significantly higher than in NCARDS patients (p = 0.001).

Neuromuscular blocker (NMB) usage during treatment was examined in CARDS and NCARDS patients. It was found that 25% of CARDS patients used NMB at some stage of treatment, while 31.3% of NCARDS patients used NMB at some stage of treatment. According to the chi-squared test, there was no statistically significant difference in the need for NMB use between the two groups (p > 0.05).

The use of vasopressor agents and inotropes (norepinephrine, adrenaline, dopamine, etc.) during treatment was also examined in CARDS patients and NCARDS patients. It was found that 78% of CARDS patients used vasopressor agents or inotropes at some stage of treatment while in NCARDS patients, 62.5% did the same. According to the Chi-squared test, there was no statistically significant difference in the need for vasopressor agents or inotropes between the two groups (p > 0.05) (Table 3).

**Table 3:** Comparison of the use of corticosteroids, NMB, and vasopressors/inotropes between COVID-19-related ARDS patients and non-COVID-19-related ARDS patients NMB: neuromuscular blocker; ARDS: acute respiratory distress syndrome; COVID-19: coronavirus disease 2019 *Chi-squared test

	COVID-19-related ARDS patients		Non-COVID-19-related ARDS patients		p-value
	Number of Patients	Percentage	Number of Patients	Percentage	
Corticosteroids					0.001
-	9	28.1	26	81.3	
+	23	71.9	6	18.8	
NMB					0.58
-	24	75.0	22	68.8	
+	8	25.0	10	31.3	
Vasopressors/inotropes					0.17
-	7	21.9	12	37.5	
+	25	78.1	20	62.5	

Six (18.8%) CARDS patients required hemodiafiltration (HDF), while 10 (31.3%) NCARDS patients required HDF. According to the chi-squared test, there was no statistically significant difference in the need for HDF between the two groups (p > 0.05). Prone positioning was applied to 20 (62.5%) CARDS patients and six (18.8%) NCARDS patients (Table 4).

**Table 4:** Comparison of the use of HDF and the administration of the prone position between COVID-19-related ARDS patients and non-COVID-19-related ARDS patients HDF: hemodiafiltration; ARDS: acute respiratory distress syndrome; COVID-19: coronavirus disease 2019 *Chi-squared test

	COVID-19-related ARDS patients		Non-COVID-19-related ARDS patients		p-value
	Number of Patients	Percentage	Number of Patients	Percentage	
HDF					0.25
-	26	81.3	22	68.8	
+	6	18.8	10	31.3	
Prone Position					0.001
-	12	37.5	26	81.3	
+	20	62.5	6	18.8	

The mean Glasgow Coma Scale (GCS) score on the first day of admission to the ICU for CARDS patients was 14.0. In NCARDS patients, the median GCS score on the first day of ICU admission was 12.4. According to the student’s t-test, the median GCS scores of CARDS patients on the first day of ICU admission were significantly higher than those of NCARDS patients (p = 0.04).

In NCARDS patients, the median heart rate on the first day of admission to the ICU was 108.1. In CARDS patients, the median heart rate on the first day of ICU admission was 94.5. According to the student’s t-test, the median heart rate of NCARDS patients on the first day of ICU admission was significantly higher than that of CARDS patients (p = 0.009).

In NCARDS patients, the median saturation level on the first day of admission to the ICU was 91.2. In CARDS patients, the median saturation level on the first day of ICU admission was 80.2. According to the student’s t-test, the median saturation levels of NCARDS patients on the first day of ICU admission were significantly higher than those of CARDS patients (p = 0.001).

The median of the highest PEEP levels assessed only in intubated patients during the follow-up was 13.8 in CARDS patients and 11.7 in NCARDS patients. According to the student’s t-test, the median PEEP levels of CARDS patients were significantly higher than those of NCARDS patients (p= 0.04) (Table 5).

**Table 5:** Comparison of some initial levels of COVID-19-related ARDS patients and non-COVID-19-related ARDS patients on their first day of admission GCS: Glasgow Coma Scale; HR: heart rate; Sat: oxygen saturation level; PEEP: positive end-expiratory pressure; ARDS: acute respiratory distress syndrome; COVID-19: coronavirus disease 2019 ^b^ Student’s t-test; ^c^ Only intubated patients were included in the evaluation

	COVID-19-related ARDS patients		Non-COVID-19-related ARDS patients		p-value
	Median	IQR	Median	IQR	
GCS^b^	14.0	2.4	12.4	3.8	0.04
HR^b^	94.5	13.1	108.1	25.6	0.009
Sat^b^	80.2	13.9	91.2	9.3	0.001
PEEP^c,b^	13.8	3.1	11.7	3.8	0.04

The median pH level on the first day of admission to the ICU for CARDS patients was 7.4. In NCARDS patients, the median pH level on the first day of ICU admission was 7.3. According to the student’s t-test, the median pH levels of CARDS patients on the first day of ICU admission were significantly higher than those of NCARDS patients (p = 0.009).

The median hemoglobin (Hgb) level on the first day of admission to the ICU for CARDS patients was 11.3. In NCARDS patients, the median Hgb level on the first day of ICU admission was 9.5. According to the student’s t-test, the median Hgb levels of CARDS patients on the first day of ICU admission were significantly higher than those of NCARDS patients (p = 0.005).

In NCARDS patients, the median neutrophil level on the first day of admission to the ICU was 12.0. In CARDS patients, the median neutrophil level on the first day of ICU admission was 8.7. According to the Wilcoxon Signed Ranks Test, the median neutrophil levels of NCARDS patients on the first day of ICU admission were significantly higher than those of CARDS patients (p = 0.03).

According to the Wilcoxon signed-rank test, the median albumin levels of CARDS patients on the first day of ICU admission (3.4) were significantly higher than those of NCARDS patients (2.8 p = 0.03) (Table 6).

**Table 6:** Comparison of some laboratory levels on the first day of admission between COVID-19-related ARDS patients and non-COVID-19-related ARDS patients ^a^ Wilcoxon signed-rank test; ^b^ Student’s t-test Hgb: hemoglobin; LAC: lactate; IQR: interquartile range; ARDS: acute respiratory distress syndrome; COVID-19: coronavirus disease 2019

	COVID-19-related ARDS patients		Non-COVID-29-related ARDS patients		p-value
	Mean	Standard Deviation	Mean	Standard Deviation	
pH^b^	7.4	0.08	7.3	0.13	0.009
Hgb^b^	11.3	2.0	9.5	28	0.005
Procalsitonin^a^	0.7	1.4	7.9	20.9	0.06
LAC^a^	2.1	1.6	1.8	1.4	0.37
Neutrophil^a^	8.5	3.1	11.3	6.1	0.03
Albumin^a^	9.6	12.3	4.2	5.4	0.03
	Median	IQR	Median	IQR	
Neutrophil^a^	8.7	5.8	12.0	9.6	0.03
Albumin^a^	3.4	1.2	2.8	1.3	0.03

The mean length of stay in the ICU for CARDS patients was 21.2 days, while it was 22.7 days for NCARDS patients. According to the Wilcoxon signed-rank test, there was no significant difference between the two groups (p = 0.52). The mean number of days to extubation for CARDS patients was 7.1 days, while it was 4.6 days for NCARDS patients. According to the Wilcoxon signed-rank test, there was no significant difference between the two groups (p = 0.08). The mean number of days to intubation for CARDS patients was 14.4 days, while it was 18.1 days for NCARDS patients. According to the Wilcoxon signed-rank test, there was no significant difference between the two groups (p = 0.44). The highest FiO2 levels applied were evaluated using a student’s t-test. The mean FiO2 level for CARDS patients was 80.9, while for NCARDS patients, the mean FiO2 level was 72.3. These levels were not significant between the two groups (p = 0.06). The patients’ APACHE II scores at their initial admission to the ICU were evaluated using a student’s t-test. The mean APACHE II score for CARDS patients was 21.9, while for NCARDS patients, the mean APACHE II score was 21.8. These levels were not significantly different between the two groups (p = 0.99) (Table 7).

**Table 7:** Comparison of ICU stay duration, extubated and intubated duration, highest FiO2 values, and APACHE II scores between COVID-19-related ARDS patients and non-COVID-19-related ARDS patients ^a^ Wilcoxon signed-rank test; ^b^ Student’s t-test FiO2: fraction of inspired oxygen; APACHE II: Acute Physiology and Chronic Health Evaluation II; ARDS: acute respiratory distress syndrome; COVID-19: coronavirus disease 2019

	COVID-19-related ARDS patients		Non-COVID-19-related ARDS patients		p-value
	Mean	Standard Deviation	Mean	Standard Deviation	
Duraiton of ICU Stay (days)	21.2	16.8	22.7	20.3	0.52
Extubated^a ^(days)	7.1	5.2	4.6	6.2	0.08
Intubated^a ^(days)	14.4	16.7	18.1	20.7	0.44
FiO_2_^b^	80.9	17.7	72.3	18.7	0.06
Apache II^b^	21.9	9.5	21.8	12.8	0.99

Mortality rates were evaluated in both groups. Among CARDS patients, 17 (53.1%) had a fatal outcome, while among NCARDS patients, 18 (56.3%) had a fatal outcome. There was no significant difference in mortality between the two groups, as determined by the Chi-square test (p > 0.05) (Table 8).

**Table 8:** Comparison of mortality in COVID-19-related ARDS patients and non-COVID-19-related ARDS patients *Chi-squared test ARDS: acute respiratory distress syndrome; COVID-19: coronavirus disease 2019

	COVID-19-related ARDS patients		Non-COVID-19-related ARDS patients		p-value
	Number of Patients	Percentage	Number of Patients	Percentage	
Mortality					0.80
-	15	46.9	14	43.8	
+	17	53.1	18	56.3	

## Discussion

In the present study, the mean age of CARDS patients was 58 years, which aligns with the findings of Haudeberg and colleagues in their study with 30 CARDS and 30 NCARDS patients, in which the mean age of CARDS patients was also 58 years [4]. However, in Haudeberg et al.'s study, the mean age of NCARDS patients was 66, while in the present study, it was 49.7 years. These results suggest that ARDS affects a wide range of age groups.

In the study conducted by Haudeberg et al., the male-to-female ratio for CARDS patients was 87%, and for NCARDS patients, it was 73%. In the present study, these ratios were 53% and 50%, respectively. The significant difference in gender distribution between the two studies suggests that gender may not be a risk factor for ARDS. Other factors such as racial differences and geographic location might play a role, emphasizing the need for further research. However, it is noteworthy that in both studies, CARDS had a higher prevalence among male patients.

In Haudeberg et al.'s study, the mean PEEP for CARDS patients was 10, while for NCARDS patients, it was 8. In the present study, these mean values were 13.8 and 11.7, respectively. The differences in these values suggest that there may be variations in clinical approaches between ICUs. However, in both studies, it is notable that CARDS patients had higher mean PEEP levels compared to NCARDS patients. This indicates that in CARDS, a more defensive approach might be necessary to achieve target saturation and tidal volume. These findings suggest that there should be differences in clinical approaches between CARDS and NCARDS [4].

In the study by Metkus et al., which included 243 CARDS and 506 NCARDS patients, they found that the mean number of days without mechanical ventilation was 13 days for both CARDS and NCARDS patients [5]. In the present study, it was 7.1 and 4.6 days for CARDS and NCARDS patients, respectively. The reason for the lower mean number of days without mechanical ventilation in our study could be due to the smaller number of patients we examined and our clinical preference for early intubation. The study also screened patients in the early stages of COVID-19 disease. The treatment procedure at that time did not include high-flow nasal cannula oxygen and non-invasive mechanical ventilation for oxygen therapy. Oxygen therapy was provided by conventional low-flow (< 15 L/min) methods and endotracheal intubation. Patients with severe ARDS (partial pressure of oxygen (PaO2/FiO2) ≤ 100, respiratory rate ≥30/minute, severe respiratory distress (dyspnea, use of extra respiratory muscles), oxygen saturation ≤90% on room air, PaO2/FiO2 < 300 in the patient receiving oxygen) was intubated when conventional low-flow oxygen was not sufficient. Additionally, in both studies, it is not specified whether the days without mechanical ventilation were assessed before or after intubation. This should be considered a limitation in both studies.

In the study conducted by Metkus et al., the mortality rate was 36% for CARDS patients and 26% for NCARDS patients [5]. In the present study, these rates were 53.1% and 56.3%, respectively. These values do not correlate with each other. The differences in the number of days without mechanical ventilation and the significantly higher vasopressor/inotrope usage rate in NCARDS patients in the present study suggest that these factors might contribute to the variation in mortality outcomes.

Chauvelot et al. conducted a study in 2020 on 10 CARDS and 13 NCARDS patients. In their study, the rate of prone positioning for CARDS patients was 62% while for NCARDS patients, it was 70% [2]. In the present study, the rate of prone positioning for CARDS patients was also 62.5%. These values correlate with each other. However, the rate of prone positioning for NCARDS patients was 18.8% in the present study. The reason for the lower rate of prone positioning in this study for NCARDS patients may be that the patients followed clinically did not require prone positioning. Also, the reason that this study found a higher rate of prone positioning for CARDS patients compared to NCARDS patients may be that our approach to prone positioning has been more widely adopted and integrated into the clinic where the study was conducted.

In the study conducted by Chauvelot et al., the mean pH levels for CARDS and NCARDS patients were 7.36 and 7.34, respectively [2]. In the present study, the mean pH level for CARDS patients was 7.40, while for NCARDS patients, it was 7.30. The reason for the lower pH level in NCARDS patients in this study may be due to the presence of metabolic issues in the patients admitted to the ICU.

In the study conducted by Hoechter and colleagues, the mean lactate levels for CARDS and NCARDS patients were 1.1 and 2.1, respectively [6]. In the present study, the mean lactate level for CARDS patients was 2.1 and for NCARDS patients was 1.8.

In the study conducted by Hoechter et al., the mean procalcitonin levels for CARDS and NCARDS patients were 0.4 and 3.5, respectively [6]. The present study found that the mean procalcitonin levels were 0.7 for CARDS patients and 7.9 for NCARDS patients. In both studies, the mean procalcitonin levels for NCARDS patients were higher than those for CARDS patients. This difference can be attributed to the fact that CARDS is a viral disease while NCARDS is more often associated with bacterial pneumonia, leading to elevated procalcitonin levels in the latter group.

In Li and colleagues’ study conducted in 2020 with 15 CARDS and 93 NCARDS patients, they examined the mean albumin levels and found that it was 4 for CARDS patients and 4.1 for NCARDS patients [7]. The present study found that the mean albumin level was 3.4 for CARDS patients and 2.8 for NCARDS patients. In the current study, the mean albumin level for NCARDS patients was lower than that for CARDS patients. This might be due to the negative acute-phase reactant effect of albumin, possibly caused by NCARDS patients developing ARDS secondary to another disease.

In Asar and colleagues’ 2021 study with 17 CARDS and 16 NCARDS patients, they examined the mean APACHE II values [8]. They found that the mean APACHE II value was 21 for both CARDS and NCARDS patients. In the present study, the mean values were 21.9 for CARDS patients and 21.8 for NCARDS patients. These values correlate with this study’s findings.

In Shi et al.'s 2021 study with 60 CARDS and 60 NCARDS patients, they found that 13% of CARDS patients and 26% of NCARDS patients required HDF [9]. In the present study, 18.8% of CARDS patients and 31.3% of NCARDS patients required HDF. These values correlate with each other. The need for HDF in NCARDS patients was higher in both studies. This suggests that ARDS is a multisystemic disease and not just a lung-specific condition.

Shi et al., in their study, found that the mean length of stay in the ICU for CARDS patients was 15 days, and for NCARDS patients, it was 17 days [9]. In the present study, the mean lengths of stay in the ICU were 21.2 days for CARDS patients and 22.7 days for NCARDS patients. The length of stay in the ICU was higher for both CARDS and NCARDS patients in the present study. However, in both studies, the mean length of stay in the ICU was higher for NCARDS patients. This could be due to the fact that ARDS tends to occur more frequently in patients who are already hospitalized.

Shi et al. examined the mean number of days patients spent on mechanical ventilation [9]. The mean for CARDS patients was 13 days and for NCARDS patients was 15 days. These values correlate with the present study which found that CARDS patients were intubated for a mean of 14.4 days while NCARDS patients had a mean of 18.8 days. The reason for the lower mean number of days spent intubated in CARDS patients may be that non-invasive methods are preferred for these patients.

In Longobardo and colleagues’ 2021 study with 16 CARDS and 32 NCARDS patients, the mortality rate for CARDS patients was 69% and for NCARDS patients, it was 72% [10]. The present study found mortality rates of 53% for CARDS patients and 56% for NCARDS patients. While these values may not correlate, in both studies, mortality was higher in NCARDS patients. This indicates that NCARDS still has a high mortality rate, and there is no specific treatment available.

The study had some limitations. Since the study was retrospective, it was based on the information in the patients' epicrisis and nurse observation. Since patients' arterial blood gas data were missing in the system, venous blood gas data were also included in our study. Other missing data in our study include advanced ventilator data (such as transpulmonary pressures or ventilation-perfusion mismatches), which could not be evaluated. Another limitation of our study is the small size of the study population. Also, the study retrospectively scanned patients followed during the COVID-19 pandemic. Since this disease had just emerged, our knowledge and experience were perhaps not sufficient. The limited number of healthcare professionals, changing working conditions and hours, and limited equipment made healthcare delivery and patient follow-up challenging in our healthcare system.

## Conclusions

This study retrospectively compared clinical, laboratory, and treatment-related data of CARDS and NCARDS patients who were admitted to the ICU in our hospital on their first admission day and their last admission day. PEEP levels, initial GCS levels, initial Hgb, albumin, and pH levels on the first admission day were significantly higher in CARDS patients. Additionally, the use of corticosteroids and prone positioning was significantly higher in CARDS patients. On the other hand, on the first admission day, heart rate, saturation, and neutrophil levels were significantly higher in NCARDS patients.

Understanding and managing ARDS is still a complex task. COVID-19 disease, on the other hand, is a deadly disease with unique symptoms and characteristics. Recognizing CARDS and identifying the differences and similarities between CARDS and NCARDS will be crucial for a better understanding, management, and treatment of this disease in the future. Similar studies should be encouraged and supported.
